# Functional loss of PKMζ in the dorsal hippocampus potentiates the time-dependent increase in false contextual fear memory and impairs spatial recognition memory in mice

**DOI:** 10.3389/fnbeh.2026.1837349

**Published:** 2026-06-11

**Authors:** Haruna Takahashi, Yuna Kanemoto, Fuka Ohnuma, Yumi Tsuneura, Tohru Matsuki, Kenjiro Seki

**Affiliations:** 1 Department of Pharmacology, School of Pharmaceutical Science, Ohu University, Koriyama, Fukushima, Japan; 2 Department of Cellular Pathology, Aichi Developmental Disability Center, Institute for Developmental Research, Kasugai, Aichi, Japan

**Keywords:** dorsal hippocampus, false contextual fear memory, contextual generalization, protein kinase M zeta (PKMζ), spatial recognition memory

## Abstract

**Introduction:**

False contextual fear memory has been attributed to a time-dependent loss of precision in contextual memory representations. In this study, we investigated the role of protein kinase M zeta (PKMζ), a key molecule in the maintenance of hippocampus-dependent long-term memory, in false contextual fear memory within the dorsal (dHPC) and ventral hippocampus (vHPC).

**Methods:**

Two weeks prior to behavioral testing, male C57BL/6J mice (7–10 weeks old) received bilateral injections of adeno-associated virus (AAV PHP.eB) into the dHPC or vHPC to induce PKMζ knockdown (PKMζ KD), overexpression of wild-type PKMζ (PKMζ WT), or kinase-inactive PKMζ (PKMζ K281R). False contextual fear memory was assessed by measuring freezing behavior in Context B at 3 and 24 h following exposure to Context A with or without unconditioned stimulus presentation [US(+) and US(−), respectively]. Spatial recognition memory was evaluated using the two-trial novel arm recognition test in the Y-maze.

**Results:**

As shown in our previous work, mice exhibited freezing in Context B after receiving a US in Context A, whereas mice that did not receive such a stimulus showed minimal freezing. These data confirmed that freezing in Context B reflects false contextual fear memory. False fear responses were evident at 3 h and were further increased at 24 h. Freezing at 24 h was markedly enhanced in dHPC PKMζ knockdown mice compared with that in AAV control-injected mice. PKMζ WT overexpression prevented the increase in freezing at 24 h, whereas PKMζ K281R overexpression mimicked the effects of PKMζ KD. Furthermore, PKMζ knockdown in the dHPC impaired spatial recognition memory, indicating that hippocampus-dependent spatial processing was disrupted. In contrast, PKMζ manipulation in the vHPC did not affect false contextual fear memory but did impair spatial recognition memory.

**Discussion:**

These findings are consistent with the possibility that functional loss of PKMζ in the dHPC affects contextual memory processes in a manner that may contribute to the time-dependent increase in false contextual fear and impaired spatial recognition memory.

## 1 Introduction

Fear generalization is an adaptive process that enables organisms to respond to potential threats in novel or ambiguous environments (Asok et al., 2018). However, excessive generalization becomes maladaptive and is a hallmark of anxiety disorders, including post-traumatic stress disorder (PTSD) (Lange et al., 2017; Lommen et al., 2024). Patients with PTSD often exhibit heightened fear responses to cues that partially resemble the traumatic context (Bian et al., 2019; Morey et al., 2020), a phenomenon that has been interpreted as reflecting reduced contextual discrimination. Acute stress is also known to impair hippocampus-dependent memory processes (de Quervain et al., 1998; Henckens et al., 2009). In rodents, diminished contextual specificity is operationalized as false contextual fear memory, wherein animals freeze in a context similar but not identical to the conditioning environment (Bae et al., 2015). Although rodent models do not fully recapitulate psychiatric disorders, they offer a valuable experimental framework for investigating the neural mechanisms limiting inappropriate fear expression. However, it remains unclear whether the disruption of long-term memory maintenance contributes to the development and escalation of false contextual fear. Therefore, understanding how contextual precision is preserved—and how its disruption permits fear responses to generalize across overlapping contexts—is essential for clarifying mechanisms that limit maladaptive generalization.

Time-dependent alterations in memory precision have been reported following fear conditioning. For instance, false fear memories can emerge as early as 3 h post-conditioning, potentially reflecting rapid degradation of the initially encoded trace (Lau et al., 2020). In our previous work, we examined whether the increase in false contextual fear could be explained solely by the passage of time (Asano et al., 2025). In that study, mice that were exposed to Context B at 3 h exhibited a marked increase in freezing when re-exposed to Context B at 24 h, and an even greater increase when re-exposed at 9 days. In contrast, when mice were not exposed to Context B at 3 h, the freezing level during their first exposure to Context B was comparable across time points: mice showed similar freezing when first exposed at 24 h and even when first exposed at 9 days, similar to the level observed at 3 h. These findings suggest that freezing during the initial encounter with Context B does not increase as a function of time, and are consistent with the interpretation that the passage of time alone is unlikely to account for the later increase in false contextual fear (Kasama et al., 2022; Asano et al., 2025). These results indicate that early exposure to a similar context is required for the progressive amplification of false contextual fear. This pattern is consistent with the idea that early post-conditioning contextual experience can interact with the original memory trace and influence subsequent fear expression (Kasama et al., 2022; Asano et al., 2025). Although false contextual fear responses can emerge within hours after conditioning, fear generalization has traditionally been examined over longer time scales. However, accumulating evidence indicates that generalization can also arise at relatively early time points, particularly following exposure to similar contexts (Fujinaka et al., 2016; Yu et al., 2021). These observations suggest that early increases in freezing in similar contexts may reflect processes that overlap with, or contribute to, the emergence of generalized fear.

Stress-related glucocorticoid receptor (GR) activation is known to impair memory by reducing synaptic strength (Rimmele et al., 2013; Battaglia et al., 2024). Additionally, we previously showed that GR activation combined with mineralocorticoid receptor (MR) inactivation contributes to the time-dependent enhancement of false fear (Asano et al., 2025). Furthermore, GR signaling has been suggested to modulate synaptic plasticity-related proteins, including protein kinase M zeta (PKMζ), potentially through the regulation of neuronal excitability and AMPA receptor trafficking (Zanca et al., 2015). PKMζ, an autonomously active protein kinase C (PKC) isoform, plays a critical role in the maintenance of long-term memory (Sacktor, 2011) by stabilizing synaptic potentiation (Serrano et al., 2008). Hippocampal PKMζ has been shown to enhance long-term potentiation and long-term contextual—but not cued—fear memory (Schuette et al., 2016). Evidence from loss-of-function studies shows that disrupting PKMζ impairs established memories, while gain-of-function manipulations have been demonstrated to enhance retention (Pastalkova et al., 2006; Sacktor, 2011; Shema et al., 2011; Patel and Zamani, 2021). These findings suggest that PKMζ-dependent maintenance mechanisms stabilize memory representations, thereby limiting their aberrant modification and constraining the spread of false fear following memory reactivation.

PKMζ is widely distributed throughout the hippocampus (Serrano et al., 2008; Hales et al., 2016), a brain structure essential for the generation and maintenance of precise contextual representations (Dimsdale-Zucker et al., 2022; Keinath et al., 2022). The dorsal hippocampus (dHPC) supports fine-grained spatial discrimination (Frankland et al., 1998; Otto and Poon, 2006), whereas the ventral hippocampus (vHPC) is more closely associated with emotional processing (Li et al., 2024; Ritger et al., 2024). The cornu ammonis (CA)2/CA3 subfields contribute to the synaptic connectivity that underlies distinct spatial encoding (Sun et al., 2017), while dentate gyrus–CA3 projections function to reduce time-dependent memory generalization (Guo et al., 2018). Furthermore, synchronous CA3 ensemble activity has been reported to facilitate event association (Oishi et al., 2019). Given these findings, in this study, we focused on the CA2/CA3 region to investigate whether PKMζ constrains false contextual fear after memory reactivation. To manipulate PKMζ, the AAV PHP.eB serotype was employed, owing to its tropism for CA2/CA3 pyramidal neurons (Okamoto et al., 2023; Alexander et al., 2024). This approach allowed for an evaluation of whether PKMζ contributes to false contextual fear memory.

## 2 Materials and methods

### 2.1 Animals and ethical considerations

All experiments were conducted in accordance with the guidelines of the Animal Care Committee of Ohu University, which granted separate approval numbers for each year of this study (2023-16, 2024-28, and 2025-22). All procedures adhered to both the university’s and ARRIVE guidelines, with rigorous efforts made to minimize distress and maintain the minimum required number of animals. The principles of laboratory animal care were strictly followed to mitigate stress. Recombinant DNA experiments received approval from the Institutional Recombinant DNA Experiments Committee of Ohu University (No. 2019004) and the Aichi Developmental Disability Center (No. 19-6). Adult male C57BL/6J mice, aged 6 weeks, were obtained from Charles River Laboratories (Yokohama, Japan) and CLEA Japan (Tokyo, Japan). Following a 1-week acclimation period, mice were randomly assigned to experimental groups. The mice were housed in a controlled environment (25 °C ± 2 °C, 12/12-h light/dark cycle [lights on at 08:00 h]) with unrestricted access to food (CE-2, CLEA Japan) and water. Behavioral data were collected and analyzed using ANY-maze software (version 6.35; Stoelting Co., Wood Dale, IL, United States).

### 2.2 Behavioral tests

Adult male C57BL/6J mice (7–10 weeks old) were randomly assigned to the respective groups for the behavioral tests. All tests were performed between 8:00 and 15:00 h. Behavioral scoring and data analysis were performed under fully blinded conditions as previously described (Asano et al., 2025). Briefly, experimenters conducting behavioral assessments were blinded to group allocation using anonymized mouse IDs. Data extraction and statistical analyses were independently performed by a separate experimenter who was also blinded to group identity, ensuring blinding across both data acquisition and analysis stages. Animals were predefined to be excluded only when viral expression was clearly outside the hippocampus. In the present experiments, all injections were confirmed to be within the intended hippocampal regions, and no animals met the predefined exclusion criteria. Sample sizes were determined based on our previous studies using the same behavioral paradigm and reflect standard practice. Variability in group sizes across experiments resulted from differences in cohort availability and viral expression success, but each experiment was conducted using an independent cohort. These factors account for the variation in sample sizes while maintaining consistency with established methodological frameworks.

#### 2.2.1 Apparatus for the contextual fear conditioning test

Contextual fear conditioning was performed following our previously established procedures (Kasama et al., 2022; Asano et al., 2025). Context A consisted of a light-gray polyvinyl chloride chamber (330 × 330 × 454 mm) equipped with a stainless-steel grid floor (24 bars, 3 mm diameter, 11 mm spacing) connected to a shock generator. Illumination was maintained at 270 lux, and the chamber was cleaned with 70% ethanol between animals. Context B was a non-shock chamber matched in overall shape but differing from Context A in multiple sensory dimensions. The walls were made of dark-brown wood (345 × 345 × 295 mm), and the floor consisted of a paper towel placed over a stainless-steel grid (17 bars, 4 mm diameter, 17.8 mm spacing) that was not connected to the shock generator, allowing mice to avoid direct contact with the grid. Illumination was reduced to 80 lux, and the chamber was cleaned with 1% acetic acid to provide a distinct olfactory cue. These differences created a perceptually distinct yet comparable environment suitable for assessing false contextual fear.

#### 2.2.2 Contextual fear conditioning test

Mice were handled for 1 min per day for 3 consecutive days before behavioral testing. Mice were habituated to Box A for 5, 60 min before the commencement of contextual fear conditioning. For the conditioning phase, mice were placed in Box A [Context A; conditioned stimulus (CS)] for 3 min. Subsequently, three electrical footshocks [1 mA, 2 s; unconditioned stimulus (US)] were delivered at 60-s intervals. The animals remained in the conditioning context for an additional 3 min following the final US delivery before being returned to their home cages. At 3 h post-conditioning [US(+)], mice were placed in Box B (Context B) for 150 s without shock, and were re-exposed to Context B for 150 s at 24 h. This experimental protocol was designated as the ABB–US(+) condition. For the control condition [ABB–US(−)], mice were placed in Context A for 360 s without receiving any US. At 3 h after Context A exposure, the mice were exposed to Context B for 150 s, followed by a 150-s re-exposure to Context B at 24 h. Chambers were cleaned with 70% ethanol between sessions to eliminate olfactory cues. Freezing behavior was automatically quantified using ANY-maze software (minimum detection duration: 1 s) (Wang et al., 2018) and expressed as the percentage of freezing time relative to the total test duration (% freezing). The central zone in Box B was defined as the middle area (172.5 × 172.5 mm). Time spent in and distance traveled within the central zone were also analyzed using ANY-maze.

#### 2.2.3 Y-maze two-trial novel arm recognition test

The Y-maze novel arm recognition test was conducted with a 1 h delay between trials. This paradigm is widely used for assessing hippocampus-dependent spatial memory and detecting impairments in memory precision (Kraeuter et al., 2019). A spatial cue was provided via a fixed light fixture illuminating a specific arm. This arm remained closed during the first trial and served as the novel arm in the second trial. The center of the Y-maze was maintained at an illuminance of 150 lux. The paradigm consisted of a 5-min encoding trial during which one arm was blocked (acquisition phase), followed by a 1-h intertrial interval, and a 1-min retrieval session (retrieval phase). The start arm remained constant across both trials; the arm accessible during the encoding trial was designated as the familiar arm, while the previously blocked arm was designated as the novel arm. At the start of the encoding trial, mice were positioned facing inward within the start arm and were allowed to freely explore both the start and familiar arms for 5 min, with the timing initiated once the subject exited the start arm. During the 1-h intertrial interval, mice were returned to their home cages while the block was removed to expose the novel arm. To eliminate olfactory cues and control for potential spatial bias, the apparatus was cleaned with 70% ethanol, and wiped dry before the retrieval trial. After the 1-h intertrial interval, mice were again placed in the start arm, facing inward, and allowed to explore all three arms of the maze for 1 min. At the end of the retrieval session, the maze was cleaned and dried before the next animal was introduced. Y-Maze spatial recognition memory was scored based on the latency to explore the novel arm and a comparison of the time spent in the novel versus familiar arms during the retrieval phase. Percentages of entries, time spent, and distance traveled were calculated separately for each of the three arms. For analysis, the value for the novel arm was compared with the mean of the two familiar arms, and this comparison was used as the index of novel arm recognition.

### 2.3 Generation of adeno-associated virus plasmids

#### 2.3.1 Plasmids

Adeno-associated virus plasmids for shRNA expression were generated using a previously described backbone (Tamura et al., 2025). Individual shRNA oligonucleotides were annealed and ligated into the plasmid through the HpaI and XhoI restriction sites. To construct AAV vectors expressing wild-type or kinase-inactive PKMζ, a synthesized gene cassette containing the human synapsin promoter-myc-PKMζ (WT)-T2A-mRFP-WPRE-poly (A) sequence (Biomatik, Ontario, Canada) was inserted into an AAV backbone plasmid (Addgene #85741) using NotI restriction sites. The kinase-inactive PKMζ mutant (K98R, equivalent to K281R in full-length PKCζ; referred to hereafter as K281R) was generated via PCR-based site-directed mutagenesis, resulting in the substitution of lysine 98 with arginine, while all other construct elements remained identical to the wild-type version. A control AAV plasmid expressing mRFP alone was generated by replacing the myc-PKMζ-T2A-mRFP cassette with an mRFP fragment using NheI and XhoI restriction enzymes. The sequences of all the plasmids were verified before AAV production.

#### 2.3.2 Knockdown validation

The shRNA sequences targeting mouse *Prkcz* mRNA were designed according to established guidelines, prioritizing optimal GC content, the avoidance of internal repeats, and the minimization of predicted off-target effects. Candidate sequences were additionally selected using the GPP Web Portal (Broad Institute). The following target sequences were used for knockdown validation: shRNA#1 (5′-TGTTCCTGGTCATCGAGTATG-3′), shRNA#2 (5′-CAAGAACGATGGTGTAGACCT-3′), and shRNA#3 (5′-CAGATGATGAGGACGTCATAA-3′). Each shRNA was cloned into the pAAV vector described above. Endogenous PKMζ expression was assessed by immunoblotting using an anti-PKMζ antibody (Abcam, #ab10897), with β-actin (Sigma-Aldrich, #A5441) serving as an internal loading control.

#### 2.3.3 AAV production

Adeno-associated virus particles were produced following previously described procedures (Kasama et al., 2022), with minor modifications. Viral genome titers were quantified by qPCR in a CFX Opus 96 system (Bio-Rad) using THUNDERBIRD Next SYBR qPCR Mix (Toyobo). Quantification was performed with a primer set—comprising a forward primer (5′-GGAACCCCTAGTGATGGAGTT-3′) and a reverse primer (5′-CGGCCTCAGTGAGCGA-3′)—targeting the AAV2 inverted terminal repeat, as previously described (Hum Gene Ther Methods. 2012; 23:18–28). Standard curves generated from serial dilutions of plasmid DNA were used to calculate viral genome copy numbers.

#### 2.3.4 Western blotting

Primary cultured mouse neurons were infected with AAVs at 3 days *in vitro* (DIV) and lysed at 7 DIV in RIPA buffer supplemented with the cOmplete EDTA-free Protease Inhibitor Cocktail (Roche). Protein samples were separated by SDS-PAGE using 10% Bis-Tris gels, transferred onto PVDF membranes (Merck), blocked for 1 h at room temperature in 5% non-fat dry milk (Cell Signaling Technology) prepared in TBST (Tris-buffered saline with 0.1% Tween-20), and incubated overnight at 4 °C with primary antibodies diluted in blocking solution. After washing, the membranes were incubated with HRP-conjugated secondary antibodies. Immunoreactive bands were visualized using a chemiluminescent substrate (FUJIFILM Wako Pure Chemical, Tokyo, Japan) and imaged with the FUSION FX system (Vilber Bio Imaging, Collégien, France).

### 2.4 AAV injection into the hippocampus

Mice were anesthetized with a mixture of medetomidine hydrochloride (0.3 mg/kg), butorphanol tartrate (5 mg/kg) (both from Wako Pure Chemical Corp.), and midazolam (4 mg/kg; Sandoz Ltd., Yamagata, Japan) to ensure complete immobilization and analgesia. Following the loss of sensation, mice were secured in a stereotaxic frame (68045, RWD Life Science, Guangdong, China). Four cranial burr holes were created using a dental drill; two were used for viral delivery, while the remaining two served as anchors for stabilizing screws. Bilateral microinjections were performed using a 32-gauge Hamilton neurosyringe (65457-02, Hamilton Co. Japan K.K., Tokyo, Japan). A volume of 1 μL was infused into each hemisphere at a rate of 0.2 μL/min. The titers for the viral vectors were as follows: AAV-RFP (control), 5 × 10^12^ vg/mL; AAV-*Prkcz*-shRNA (PKMζ knockdown), 1.55 × 10^13^ vg/mL; AAV-PKMζ WT (PKMζ overexpression), 3 × 10^13^ vg/mL; and AAV-PKMζ K281R (encoding a kinase-inactive mutant), 6 × 10^13^ vg/mL. Bilateral injections were targeted to the CA2/CA3 regions of the hippocampus according to the Paxinos mouse brain atlas (Williams, 2000; Franklin and Paxinos, 2008). The coordinates for the dorsal CA2/CA3 were as follows: anteroposterior (AP), −1.46 mm; lateral (L), ±1.4 mm from bregma; and DV, −1.6 mm. For the ventral CA2/CA3, the coordinates were: AP, −2.92 mm; L, ±2.8 mm from bregma; and DV, −3.5 mm. Following AAV infusion, the neurosyringe remained *in situ* for at least 5 min to minimize the leakage of the viral suspension along the needle track (Yang et al., 2016). Next, both holes were sealed with dental cement (GC Unifast II, GC Dental Products Corp., Tokyo, Japan) secured by stabilizing screws. After recovery from anesthesia, mice were returned to their home cages for 14 days before behavioral tests.

### 2.5 Immunohistochemical verification of viral injection sites

To verify viral injection sites, mice were anesthetized with a mixture of medetomidine hydrochloride (0.3 mg/kg), butorphanol tartrate (5 mg/kg), and midazolam (4 mg/kg). Transcardial perfusion was performed with ice-cold 0.1 M PBS, followed by 4% paraformaldehyde (PFA) in 0.1 M PBS for tissue fixation. Following removal, brains were post-fixed and embedded in OCT compound (Sakura Finetek Japan Co., Ltd., Tokyo, Japan), and then frozen at −80°C for 30 min. Coronal sections (20 μm) were obtained using a cryostat at −20°C (Leica CM1100, Leica Microsystems, Wetzlar, Germany) and mounted onto poly-L-lysine-coated slides (S7441, Matsunami Glass Ind., Ltd., Osaka, Japan). After incubation for 30 min in PBS containing 0.2% Tween-20 and 10% normal donkey serum (565 73631, FUJIFILM Wako Pure Chemical Corp.), sections were incubated for 60 min at room temperature with primary antibodies diluted in Can Get Signal Immunostain Solution A (NKB 501, TOYOBO CO., LTD., Osaka, Japan). RFP was detected using mouse monoclonal anti-RFP antibody (M155-3, MBL Life Science, 1:200) or rabbit polyclonal anti-RFP antibody (PM005, MBL Life Science, 1:200); and PKCζ/PKMζ was detected with mouse monoclonal anti-PKCζ/PKMζ antibody (SC-17781, Santa Cruz Biotechnology, 1:500) or rabbit polyclonal anti-PKCζ/PKMζ antibody (ab108970, Abcam, 1:500). After three washes in PBS, sections were incubated for 2 h at room temperature with Alexa Fluor-conjugated secondary antibodies. Depending on the primary antibodies used, sections were treated with either a mouse-specific combination (Alexa Fluor 488 donkey anti-mouse IgG, ab15015, 1:200; and Alexa Fluor 555 donkey anti-mouse IgG, ab150106, 1:200) or a rabbit-specific combination (Alexa Fluor 488 donkey anti-rabbit IgG, ab150105, 1:200; and Alexa Fluor 555 donkey anti-rabbit IgG, ab150074, 1:200). Nuclear counterstaining was performed with TO-PRO-3 (Thermo Fisher, 1:5000) for 15 min at room temperature. Sections were washed in PBS containing 0.2% Tween-20 and mounted with an anti-fade medium. Fluorescence images were acquired using a confocal laser scanning microscope (LSM 510, Carl Zeiss, Oberkochen, Germany) employing identical imaging parameters for all animals.

### 2.6 Data analysis

Statistical analyses were performed using EZR (Easy R, version 1.38; Saitama Medical Center, Jichi Medical University, Saitama, Japan) (Kanda, 2013) and BellCurve for Excel (version 3.20; Social Survey Research Information Co., Ltd, Tokyo, Japan) (Kimura et al., 2020). Comparisons between two independent groups were conducted using Student’s *t*-tests. Comparisons among three or more independent groups were analyzed using one-way ANOVA followed by Bonferroni’s *post-hoc* test for multiple comparisons. Freezing levels in Context A were analyzed using a three-way repeated-measures ANOVA with US and Gene as between-subject factors and Minute as a within-subject factor, followed by Bonferroni’s *post-hoc* test when appropriate. False contextual fear responses (freezing time, %) in Context B were analyzed using a three-way repeated-measures ANOVA with US and Gene as between-subject factors and Time (3 vs. 24 h) as a within-subject factor, followed by Bonferroni’s *post-hoc* test when appropriate. To further examine temporal changes in freezing levels in Context B, differences between the 3 and 24 h time points were analyzed using a two-way ANOVA with Time and Group as factors, followed by Bonferroni’s *post-hoc* test. Error bars in bar graphs represent the standard error of the mean. Statistical significance was defined as *p* < 0.05 or *p* < 0.01. Statistical assumptions were verified prior to analysis. Normality was assessed using the Shapiro-Wilk test, and homogeneity of variance was evaluated using Levene’s test. Effect sizes for ANOVA are reported as partial eta squared ($\eta_{\text{p}}^{2}$), and Cohen’s d was calculated for pairwise comparisons. The interpretation of $\eta_{\text{p}}^{2}$ effect sizes for one-way ANOVA was based on commonly used thresholds: small effect, $\eta_{\text{p}}^{2}$ ≤ 0.03; medium/moderate effect, 0.03 < $\eta_{\text{p}}^{2}$ < 0.10; large effect, 0.10 ≤ $\eta_{\text{p}}^{2}$ < 0.20; very large effect, $\eta_{\text{p}}^{2}$ ≥ 0.20 (Colquhoun, 2014). Cohen’s d (|d|) for unpaired *t*-tests was interpreted according to commonly used thresholds in mouse neurobehavioral studies: small effect, |d| ≤ 0.5; medium/moderate effect, 0.5 < |d| < 1.0; large effect, 1.0 ≤ |d| < 1.5; very large effect, |d| ≥ 1.5 (Labots et al., 2016). Change scores (24–3 h) were analyzed to quantify the magnitude of within-subject time-dependent changes, providing a complementary measure to repeated-measures ANOVA by directly capturing temporal shifts in freezing behavior independent of baseline variability. Sample sizes were based on our previous studies using the same behavioral paradigm and are consistent with standard practice in behavioral neuroscience. A formal a priori power analysis was not performed; however, the observed effect sizes were consistent with those reported in previous studies using the same experimental framework, indicating that the experiments were sufficiently sensitive to detect the primary effects of interest.

## 3 Results

### 3.1 Validation of PKMζ knockdown in the dHPC and its effect on false contextual fear memory

**FIGURE 1:**
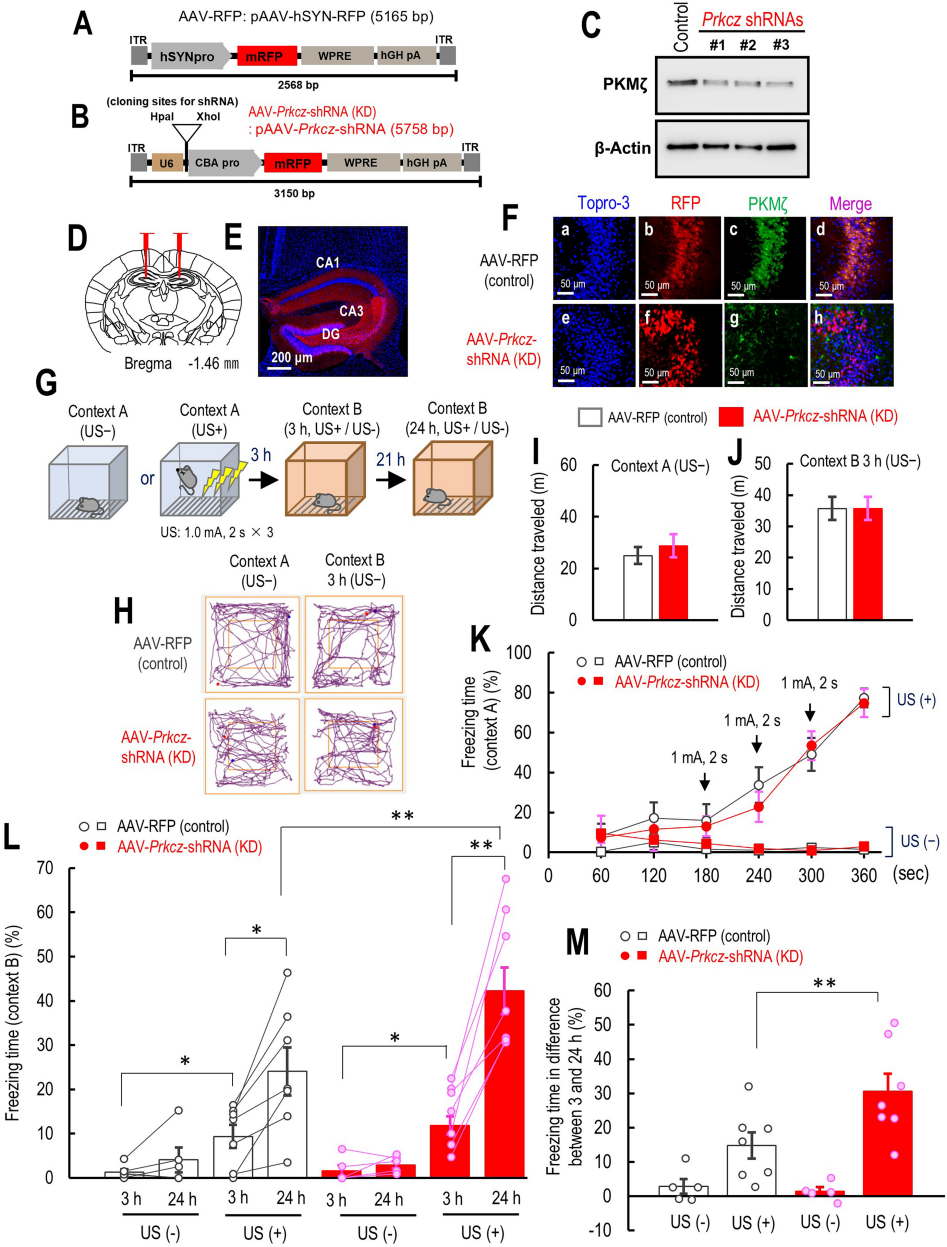
**(A)** Schematic of adeno-associated virus (AAV) constructs used for protein kinase M zeta (PKMζ) knockdown (AAV-*Prkcz*-shRNA and AAV-RFP). **(B)** Diagram of the AAV-*Prkcz*-shRNA vector. **(C)** Immunoblot showing reduced PKMζ protein levels in primary cultured neurons transduced with AAV-*Prkcz*-shRNA. **(D)** Schematic of bilateral AAV injections into the dorsal hippocampus (dHPC). **(E)** Representative red fluorescent protein (RFP) expression in CA3 pyramidal neurons and dentate gyrus granule cells following AAV-PHP.eB transduction. **(F)** Representative immunohistochemical images of PKMζ expression in CA3 pyramidal neurons. **(G)** Behavioral schedule for contextual fear conditioning and testing. **(H)** Representative locomotor traces in Context A and Context B under US(–) conditions. **(I,J)** Locomotor activity in Context A and Context B [control US(–), *n* = 5; PKMζ KD US(–), *n* = 5]. **(K)** Freezing behavior during conditioning in Context A [control US(- ), *n* = 5; PKMζ KD US(- ), *n* = 5; control US(+), *n* = 7; PKMζ KD US(+), *n* = 7]. **(L)** Freezing levels in Context B at 3 h and 24 h post-conditioning (same group sizes as in K). **(M)** Change in freezing (24–3 h). *p* < 0.05, *p* < 0.01 (Bonferroni-corrected *post-hoc* tests). *p < 0.05, **p< 0.01.

The AAV constructs used in this study are shown in Figures 1A, B. AAV-*Prkcz*-shRNA was designed to reduce PKMζ expression (PKMζ KD virus), while AAV-RFP served as the control. Consistent with this design, compared with the control virus, the PKMζ KD virus significantly reduced PKMζ protein levels in primary cultured neurons (Figure 1C and Supplementary Figures 1A,B). Subsequently, control and PKMζ KD viruses were bilaterally injected into the dHPC of mice (Figure 1D). RFP signals were observed in CA3 pyramidal neurons and dentate gyrus granule cells, whereas CA1 pyramidal neurons remained unlabeled; the AAV-PHP.eB serotype showed strong tropism for neurons in the CA3 and dentate gyrus neurons (Figure 1E). Accordingly, PKMζ expression in CA3 pyramidal neurons was markedly reduced in PKMζ KD mice relative to that seen in control mice (Figure 1F). In this paradigm, mice were exposed to Context A with or without US and were subsequently tested in Context B at 3 and 24 h post-conditioning (Figure 1G). In the absence of an US, both control and PKMζ KD mice freely explored the entire arena in Context A and Context B, as shown in the representative locomotor activity traces in Figure 1H; these data indicated that PKMζ KD did not impair general exploratory behavior. Consistent with this observation, when mice were not exposed to an US in Context A, locomotor activity did not differ between control and PKMζ KD mice in either Context A or Context B (Context A: *t*(8) = 0.70, *p* = 0.504, Cohen’s d = 0.44; Context B: *t*(8) = 0.35, *p* = 0.737, Cohen’s *d* = 0.22; Figures 1I, J). A three-way repeated-measures ANOVA (US × Gene × Minute; Gene: control vs. PKMζ KD) revealed no significant effects of Gene in Context A, including the Gene × Minute interaction [*F*(5,100) = 0.38, *p* = 0.864, η^2^_*p*_ = 0.02; Figure 1K] and the main effect of Gene [*F*(1,20) = 0.008, *p* = 0.93, η^2^_*p*_ < 0.01]. These findings suggested that the knockdown of PKMζ in the dHPC did not affect fear acquisition during conditioning. In contrast, a three-way repeated-measures ANOVA (US × Gene × Time; Gene: control vs. PKMζ KD) revealed a significant US × Gene × Time interaction for freezing levels in Context B [*F*(2,20) = 4.58, *p* < 0.05, η^2^_*p*_ = 0.31; Figure 1L]. Bonferroni-corrected *post hoc* comparisons showed that control mice exhibited significantly increased freezing in the US(+) condition than in the US(−) condition at the 3 h time point (*p* < 0.05), indicating that false contextual fear memory was induced. Freezing further increased at 24 h compared with that at 3 h in the US(+) condition (*p* < 0.05), suggesting that the false fear response was strengthened over time. Similarly, PKMζ KD mice showed significantly increased freezing in the US(+) condition than in the US(−) condition at 3 h (*p* < 0.05), and freezing further increased from 3 to 24 h (*p* < 0.05). In addition, freezing at 24 h in the US(+) condition was significantly elevated in PKMζ KD mice compared with control mice (*p* < 0.01; Figure 1L). A one-way ANOVA of the change in freezing (24 h–3 h) revealed a significant group effect [*F*(3,23) = 11.68, *p* < 0.01, η^2^_*p*_ = 0.60; Figure 1M). Bonferroni-corrected *post hoc* comparisons indicated that the increase in freezing from 3 to 24 h was significantly greater in PKMζ KD mice than in control mice (*p* < 0.01). These data demonstrated that the knockdown of PKMζ KD in the dHPC facilitates the time-dependent increase in false contextual fear memory.

### 3.2 Effects of PKMζ WT and K281R overexpression in the dHPC on false contextual fear memory

**FIGURE 2:**
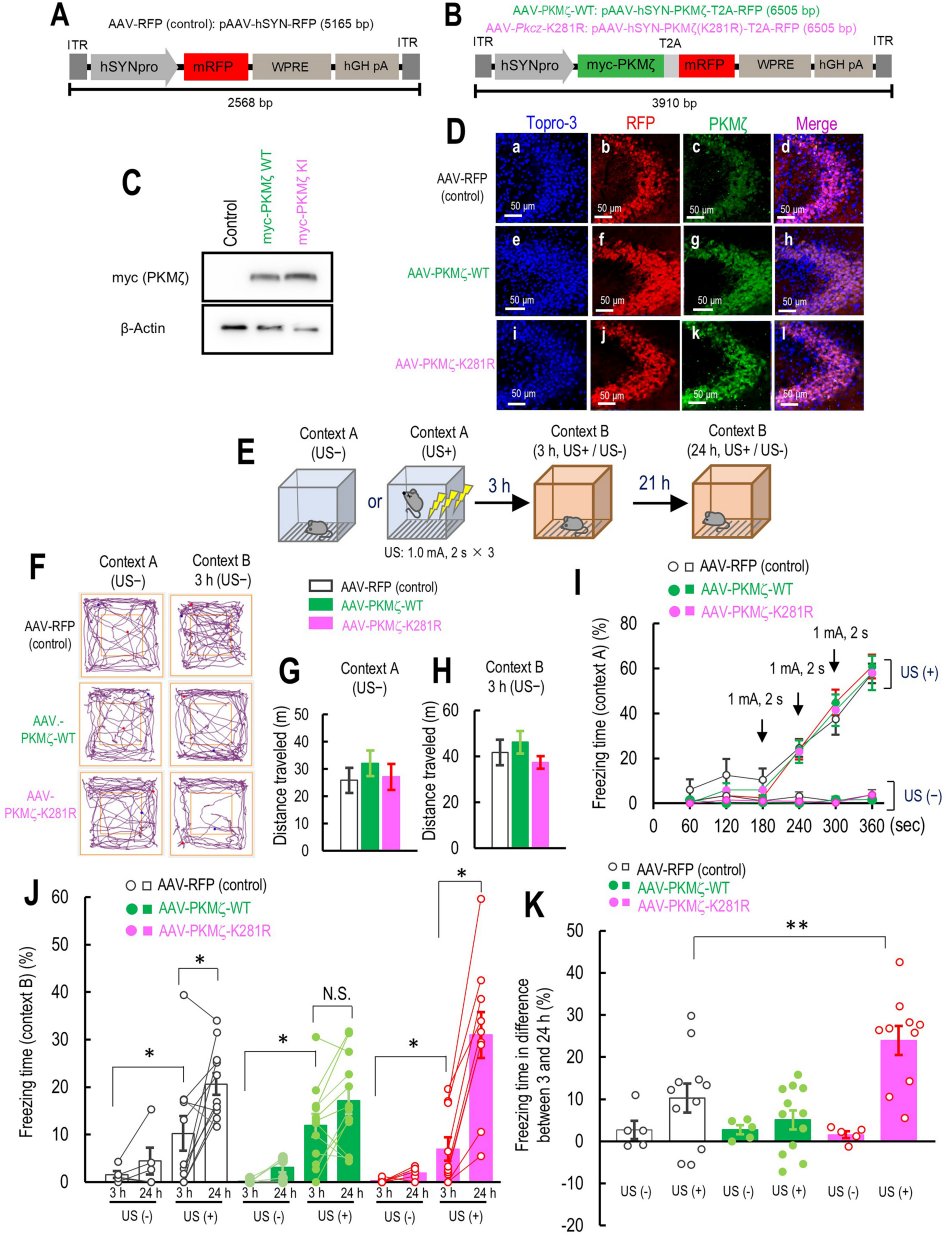
**(A)** Schematic of adeno-associated virus (AAV) constructs used for protein kinase M zeta (PKMζ) overexpression (AAV-PKMζ-WT, AAV-PKMζ-K281R, and AAV-RFP). **(B)** Schematic of bilateral AAV injections into the dorsal hippocampus (dHPC). **(C)** Immunoblot showing increased PKMζ protein levels in primary cultured neurons transduced with AAV-PKMζ-WT or AAV-PKMζ-K281R. **(D)** Representative images of PKMζ expression in CA3 pyramidal neurons following *in vivo* AAV injections. **(E)** Behavioral schedule for contextual fear conditioning and testing. **(F)** Representative locomotor traces in Context A and Context B under US(–) conditions. **(G,H)** Locomotor activity in Context A and Context B [control US(–), *n* = 5; PKMζ WT US(–), *n* = 5; PKMζ K281R US(–), *n* = 5]. **(I)** Freezing behavior during conditioning in Context A [control US(–), *n* = 5; PKMζ WT US(–), *n* = 5; PKMζ K281R US(–), *n* = 5; control US(+), *n* = 13; PKMζ WT US(+), *n* = 12; PKMζ K281R US(+), *n* = 15]. **(J)** Freezing levels in Context B at 3 and 24 h post-conditioning [control US(–), *n* = 5; PKMζ WT US(–), *n* = 5; PKMζ K281R US(–), *n* = 5; control US(+), *n* = 11; PKMζ WT US(+), *n* = 12; PKMζ K281R US(+), *n* = 10]. **(K)** Change in freezing (24 h–3 h). *p* < 0.05, *p* < 0.01 (Bonferroni-corrected *post-hoc* tests). *p < 0.05, **p< 0.01.

We next investigated whether the overexpression of PKMζ WT or the kinase-inactive mutant K98R in the dHPC influences false contextual fear memory. The AAV constructs used for overexpression are shown in Figures 2A, B. AAV-PKMζ WT and AAV-PKMζ K281R were designed to elevate PKMζ expression (PKMζ WT and PKMζ K281R viruses, respectively), with AAV-RFP serving as the control. As expected, both PKMζ WT and PKMζ K281R viruses markedly increased PKMζ protein levels in primary cultured neurons compared with those observed with the control virus (Figure 2C and Supplementary Figures 1C,D). Following bilateral injections of control, PKMζ WT, or PKMζ K281R viruses into the dHPC, PKMζ expression was detected in CA3 pyramidal neurons in all groups (Figure 2D), confirming that transduction was successful. In this behavioral paradigm, mice were exposed to Context A with or without US and subsequently tested in Context B at 3 and 24 h post-conditioning (Figure 2E). In the US(−) condition, control mice, PKMζ WT mice, and PKMζ K281R mice freely explored the entire arena in both contexts, as shown by representative locomotor traces (Figure 2F); these data indicated that neither form of PKMζ overexpression affected general exploratory behavior. Furthermore, one-way ANOVA revealed that there were no differences in locomotor activity among the three groups in either Context A or Context B [Context A: *F*(2,14) = 0.50, *p* = 0.618, η^2^_*p*_ = 0.07; Context B: *F*(2,14) = 1.20, *p* = 0.33, η^2^_*p*_ = 0.15; Figures 2G, H]. Meanwhile, a three-way repeated-measures ANOVA (Conditioning × Gene × Time; Gene: control *vs*. PKMζ WT vs. PKMζ K281R) revealed no significant Conditioning × Gene × Time interaction [*F*(10,220) = 0.24, *p* = 0.99, η^2^_*p*_ = 0.01; Figure 2I]. There was also no significant Gene × Time interaction [*F*(10,220) = 0.33, *p* = 0.97, η^2^_*p*_ = 0.02] and no significant main effect of Gene [*F*(2,44) = 0.17, *p* = 0.84, η^2^_*p*_ = 0.01]. These findings indicated that neither PKMζ WT nor PKMζ K281R overexpression in the dHPC influenced fear acquisition during conditioning. In contrast, analysis of freezing in Context B revealed a significant US × Gene × Time interaction (Figure 2J). Bonferroni-corrected *post hoc* comparisons confirmed that US exposure in Context A induced significant freezing in Context B at 3 h in all groups (control, PKMζ WT, and PKMζ K281R; all *p* < 0.05 vs. the respective US(−) groups); these data implied that neither construct affected the initial formation of false contextual fear memory. In control mice, freezing in Context B was significantly increased at 24 h compared with that at 3 h (*p* < 0.05), reflecting the expected time-dependent increase in false fear memory. A similar increase was observed in PKMζ K281R mice, where freezing at 24 h was significantly elevated compared with that in control mice (*p* < 0.05; Figure 2J). These data demonstrated that the loss of PKMζ kinase activity strengthens the formation of false contextual fear memory, consistent with the phenotype observed in PKMζ KD mice. Conversely, PKMζ WT overexpression abolished this increase, resulting in comparable freezing levels at 24 and 3 h (*p* = 0.823; Figure 2J). This finding suggests that functional PKMζ prevents the time-dependent enhancement of false contextual fear memory. Supporting these findings, a one-way ANOVA of the change in freezing levels (24−3 h) revealed a significant group effect [*F*(5,46) = 8.30, *p* < 0.01, η^2^_*p*_ = 0.47; Figure 2K]. Bonferroni-corrected comparisons showed that the increase in freezing from 3 to 24 h was significantly greater in PKMζ K281R mice than in control mice (*p* < 0.01), further implying that the inactivation of PKMζ kinase activity in the dHPC promotes the time-dependent strengthening of false contextual fear memory.

### 3.3 The role of dorsal hippocampal PKMζ in hippocampus-dependent spatial memory

**FIGURE 3:**
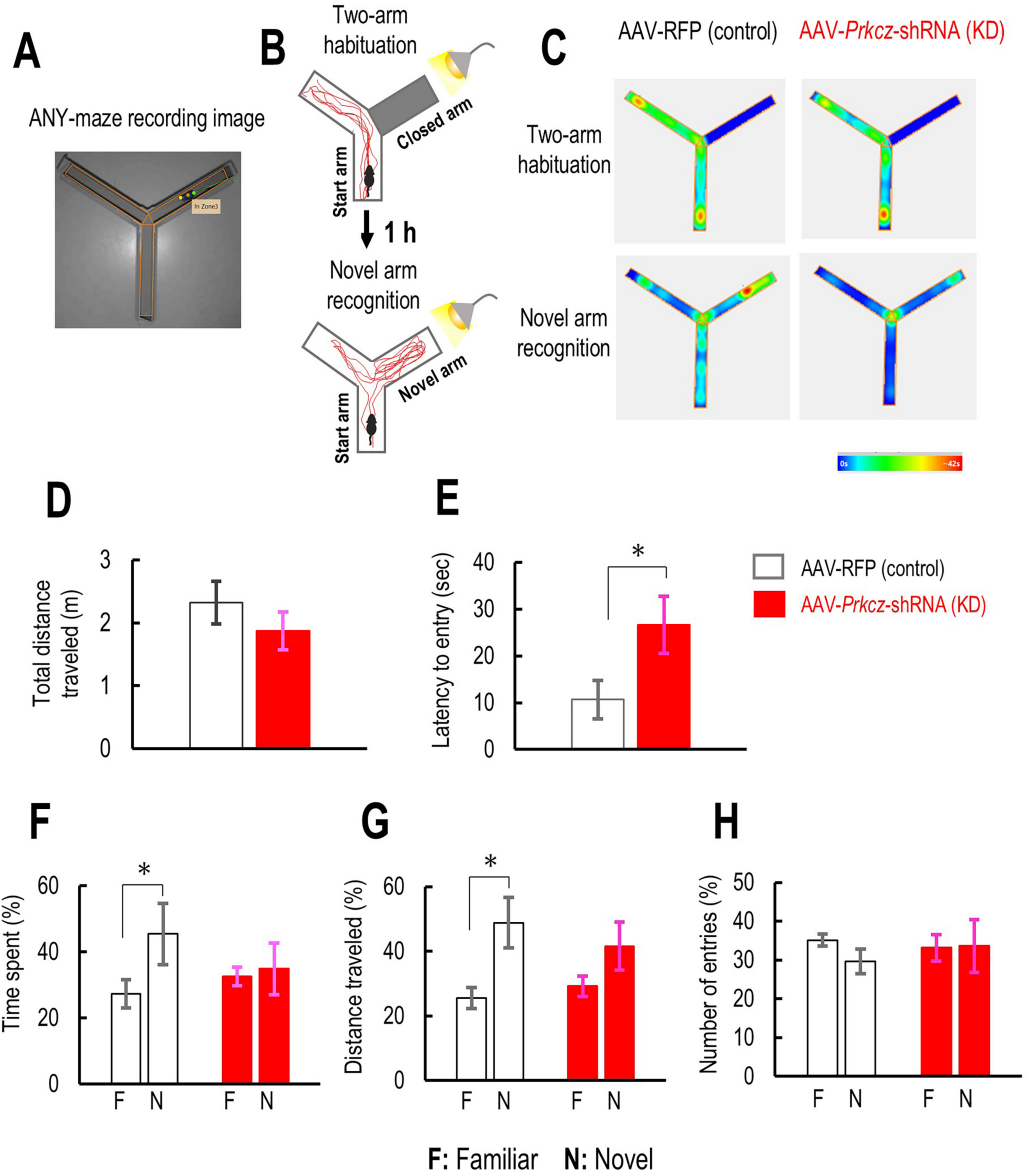
**(A)** Representative image of a mouse exploring the Y-maze (ANY-maze software). **(B)** Schematic of the Y-maze novel arm recognition paradigm. **(C)** Representative heat maps of exploration time during the two-arm (upper) and three-arm (lower) phases. **(D)** Total distance traveled during the three-arm phase (control, *n* = 8; PKMζ KD, *n* = 8). **(E)** Latency to enter the novel arm during the three-arm phase (*n* = 8 per group). **(F)** Percentage of time spent in each arm during the three-arm phase (*n* = 8 per group). Values for the two familiar arms were averaged. **(G)** Percentage of total distance traveled in each arm during the three-arm phase (*n* = 8 per group). Values for the two familiar arms were averaged. **(H)** Number of entries into each arm during the three-arm phase (*n* = 8 per group). *p* < 0.05 (Student’s *t*-test). *p < 0.05, **p< 0.01.

To determine how PKMζ in the dHPC contributes to hippocampus-dependent spatial memory, we assessed the performance of PKMζ KD mice in the Y-maze novel arm recognition test. A representative image of a mouse exploring the Y-maze during the task, captured using ANY-maze software, is shown in Figure 3A. The behavioral procedure is illustrated in Figure 3B. Mice first explored the maze while access to one arm was blocked (two-arm phase) and were subsequently reintroduced with all three arms accessible (three-arm phase) to evaluate novel arm recognition. Representative heat maps of the exploration time are shown in Figure 3C. During the two-arm phase, both control and PKMζ KD mice explored the two available arms to similar extents, resulting in the uniform distribution of dwell times. Meanwhile, during the three-arm phase, control mice preferentially explored the novel arm, as reflected by the prominent red signal. In comparison, PKMζ KD mice showed a more uniform distribution of dwell times across all three arms, suggesting that PKMζ KD impaired novel arm recognition. The total distance traveled during the three-arm phase did not differ between control and PKMζ KD mice [*t*(11) = 0.997, *p* = 0.338, Cohen’s *d* = 0.57; Figure 3D], indicating that PKMζ insufficiency did not affect general locomotor activity. We next examined the latency to enter the novel arm during the three-arm phase. Compared with control animals, PKMζ KD mice required significantly more time to enter the previously restricted arm [*t*(11) = 2.345, *p* < 0.05, Cohen’s *d* = 1.35; Figure 3E], further supporting the conclusion that the knockdown of PKMζ impaired novel arm recognition. One-way ANOVA revealed significant group differences in both the time spent and the distance traveled in the arms [time: *F*(3,27) = 2.620, *p* < 0.05, η^2^_*p*_ = 0.23; distance: *F*(3,27) = 3.159, *p* < 0.05, η^2^_*p*_ = 0.26; Figures 3F, G]. Bonferroni-corrected *post hoc* comparisons showed that control mice spent significantly more time in, and traveled a greater distance within, the novel arm relative to the familiar arms (both *p* < 0.05). Conversely, PKMζ KD mice showed no significant differences in the time spent or distance traveled between the novel and familiar arms (both *p* = 1.000; Figures 3F, G). Furthermore, the number of entries into the novel and familiar arms was similar for both control and PKMζ KD mice [*F*(3,27) = 0.2997, *p* = 0.8252, η^2^_*p*_ = 0.03; Figure 3H], indicating that the downregulation of PKMζ did not influence exploratory drive. Together, these findings demonstrated that functional loss of PKMζ in the dHPC impairs hippocampus-dependent spatial memory while enhancing the time-dependent increase in false contextual fear memory.

### 3.4 PKMζ knockdown in the vHPC does not affect false contextual fear memory

**FIGURE 4:**
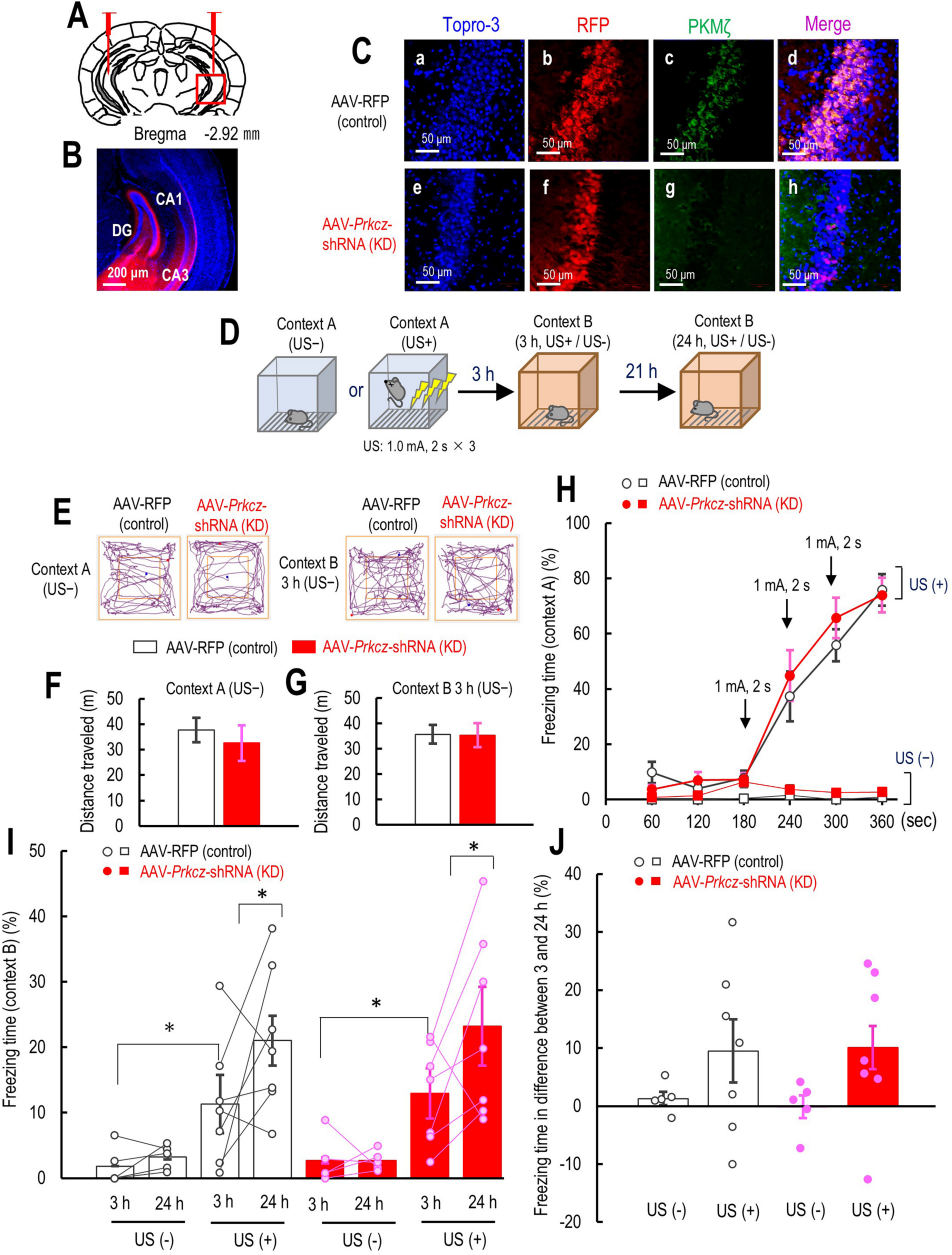
**(A)** Schematic of bilateral injections of AAV-*Prkcz*-shRNA or AAV- RFP into the ventral hippocampus (vHPC). **(B)** Representative red fluorescent protein (RFP) expression in CA3 pyramidal neurons and dentate gyrus granule cells of the vHPC. **(C)** Representative images showing PKMζ expression in vHPC CA3 pyramidal neurons. **(D)** Behavioral schedule for contextual fear conditioning and testing. **(E)** Representative locomotor traces in Context A and Context B under US(–) conditions. **(F,G)** Locomotor activity in Context A and Context B [control US(–), *n* = 5; PKMζ KD US(–), *n* = 5]. **(H)** Freezing behavior during conditioning in Context A (control US–), *n* = 5; PKMζ KD US(–), *n* = 5; [control US(+), *n* = 7; PKMζ KD US(+), *n* = 7]. **(I)** Freezing levels in Context B at 3 and 24 h post-conditioning (same group sizes as in panel **(H)**. **(J)** Change in freezing (24–3 h). *p* < 0.05 (Bonferroni-corrected *post-hoc* tests). *p < 0.05, **p< 0.01.

To verify the efficiency of PKMζ knockdown in the vHPC, we examined PKMζ expression following bilateral AAV-*Prkcz* shRNA injection. AAV-RFP and AAV-*Prkcz* shRNA signals were detected in CA3 pyramidal neurons and dentate gyrus granule cells, but not in CA1 pyramidal neurons, confirming the selective transfection pattern of the AAV-PHP.eB serotype (Figures 4A, B). In shRNA-injected mice, PKMζ expression in vHPC CA3 pyramidal neurons was significantly reduced compared with that in control animals (Figure 4C). This decrease in PKMζ expression did not affect locomotor activity in either Context A or Context B [Context A: *t*(8) = 0.606, *p* = 0.562, Cohen’s *d* = 0.38; Context B: *t*(8) = 1.85, *p* = 0.213, Cohen’s *d* = 1.16; Figures 4D–F, G]. Furthermore, a three-way repeated-measures ANOVA (Conditioning × Gene × Time) revealed no significant Conditioning × Gene × Time interaction [*F*(5,100) = 0.53, *p* = 0.75, η^2^_*p*_ = 0.03; Figure 4H]. There was also no significant Gene × Time interaction [*F*(5,100) = 0.58, *p* = 0.71, η^2^_*p*_ = 0.03] and no significant main effect of Gene [*F*(1,20) = 0.64, *p* = 0.43, η^2^_*p*_ = 0.03]. These findings indicated that PKMζ knockdown in the vHPC did not affect fear acquisition during conditioning. We next examined whether PKMζ knockdown in the vHPC affected false contextual fear responses. A three-way repeated-measures ANOVA (US × Gene × Time) revealed that there was no significant interaction among these factors relating to freezing levels in Context B at either 3 or 24 h post-conditioning [*F*(1,20) = 0.143, *p* = 0.710, η^2^_*p*_ = 0.007; Figure 4I]. However, a two-way repeated-measures ANOVA (US × Time) confirmed that Context B elicited false fear responses at 3 h following US exposure in Context A [*F*(1,20) = 3.66, *p* < 0.05, η^2^_*p*_ = 0.15]. Bonferroni-corrected *post-hoc* tests further showed that both control and PKMζ KD mice exhibited a significant increase in false fear responses at 24 h compared with those at 3 h (both *p* < 0.05; Figure 4I). Importantly, the magnitude of the increase at 24 h in PKMζ KD mice was comparable to that of controls (*p* = 1.00; Figure 4I). Furthermore, analysis of the change in freezing levels (24 h–3 h) revealed no significant difference between groups [two-way ANOVA, US × Gene: *F*(1,19) = 0.256, *p* = 0.619, η^2^_*p*_ = 0.013; Figure 4J]. These results indicated that PKMζ knockdown in the vHPC does not contribute to the formation or the time-dependent increase in false contextual fear memory.

### 3.5 Effects of PKMζ WT and K281R overexpression in the ventral hippocampus on false contextual fear memory

**FIGURE 5:**
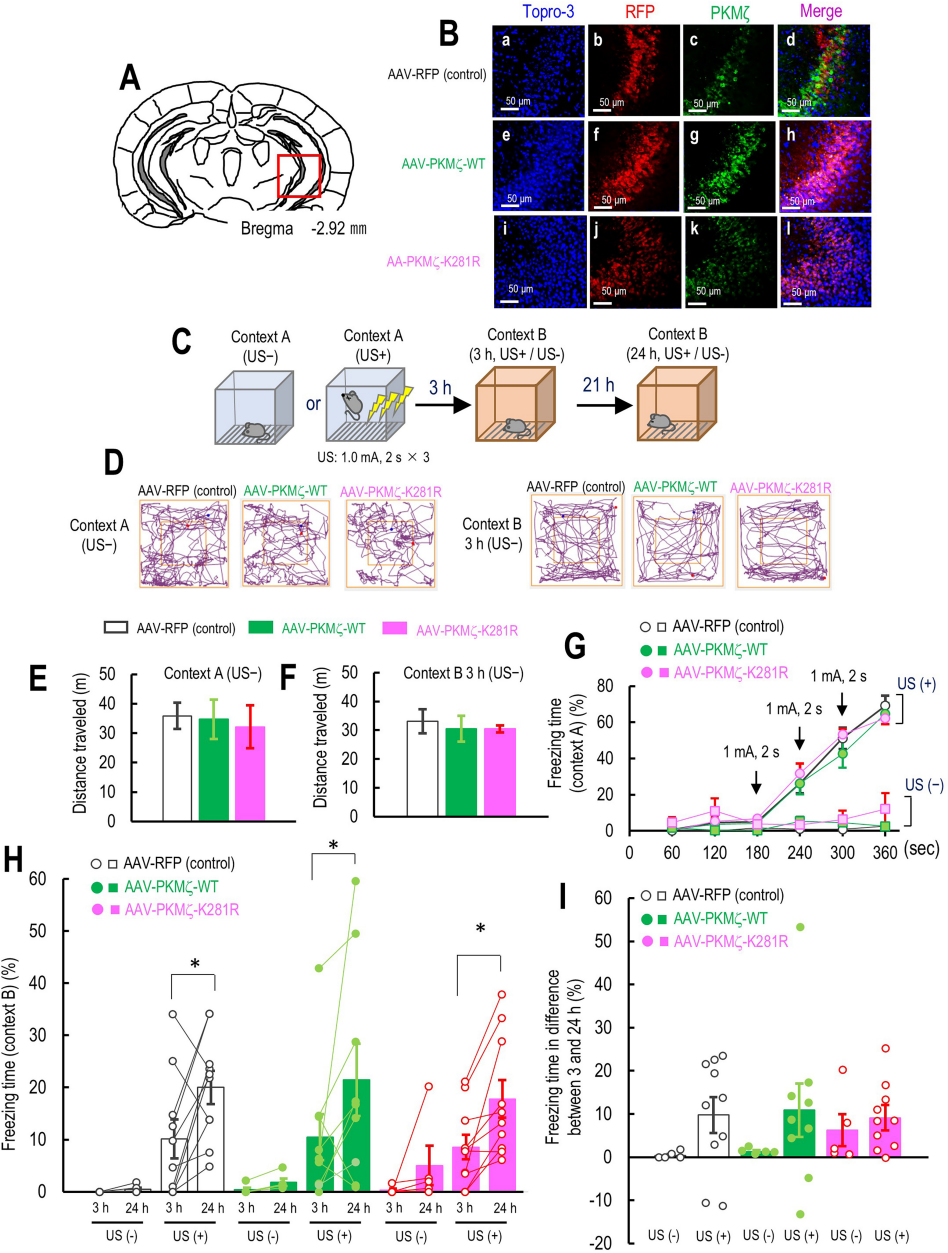
**(A)** Schematic of bilateral injections of AAV-PKMζ-WT, AAV-PKMζ-K281R, or AAV-RFP into the ventral hippocampus (vHPC). **(B)** Representative protein kinase M zeta (PKMζ) expression in CA3 pyramidal neurons across experimental groups. **(C)** Behavioral schedule for contextual fear conditioning and testing. **(D)** Representative locomotor traces in Context A and Context B under US(–) conditions. **(E,F)** Locomotor activity in Context A and Context B [control US(–), *n* = 5; PKMζ WT US(–), *n* = 5; PKMζ K281R US(–), *n* = 5]. **(G)** Freezing behavior during conditioning in Context A [control US(–), *n* = 5; PKMζ WT US(–), *n* = 5; PKMζ K281R US(–), *n* = 5; control US(+), *n* = 10; PKMζ WT US(+), *n* = 9; PKMζ K281R US(+), *n* = 10]. **(H)** Freezing levels in Context B at 3 and 24 h post-conditioning [same group sizes as in panel **(G)**]. **(I)** Change in freezing (24–3 h). *p* < 0.05 (Bonferroni-corrected *post-hoc* tests). *p < 0.05, **p< 0.01.

We next examined whether overexpressing PKMζ WT or the kinase-inactive K281R mutant in the vHPC influenced false contextual fear memory. Following bilateral injections of AAV-RFP, AAV-PKMζ WT, or AAV-PKMζ K281R into the vHPC, robust PKMζ expression was detected in CA3 pyramidal neurons in both overexpression groups (Figures 5A, B). Neither PKMζ WT nor K281R overexpression affected locomotor activity in either context [Context A: one-way ANOVA, *F*(2,14) = 0.092, *p* = 0.913, η^2^_*p*_ = 0.01; Context B: *F*(2,14) = 0.289, *p* = 0.754, η^2^_*p*_ = 0.04; Figures 5C–F, G]. During conditioning in Context A, a three-way repeated-measures ANOVA (Conditioning × Gene × Time) revealed no significant Conditioning × Gene × Time interaction [*F*(10,220) = 1.31, *p* = 0.23, η^2^_*p*_ = 0.06] and no Gene × Time interaction [*F*(10,220) = 1.04, *p* = 0.41, η^2^_*p*_ = 0.05; Figure 5G]. The main effect of Gene approached but did not reach significance [*F*(2,44) = 3.19, *p* = 0.051, η^2^_*p*_ = 0.13]. These results indicated that neither PKMζ WT nor K281R overexpression in the vHPC significantly affects fear acquisition. Regarding false contextual fear responses in Context B, a three-way repeated-measures ANOVA revealed no significant US × Gene × Time interaction relating to freezing levels at either 3 or 24 h post-conditioning [*F*(2,38) = 0.202, *p* = 0.818, η^2^_*p*_ = 0.01; Figure 5H]. *Post-hoc* Bonferroni tests confirmed that US exposure in Context A induced significant freezing in Context B at 3 h in all groups (control, PKMζ WT, and PKMζ K281R; all *p* < 0.05 versus the respective non-US groups), indicating that the formation of false contextual fear memory was not disrupted. In control mice, freezing in Context B was significantly increased at 24 h relative to that at 3 h (*p* < 0.05), thus demonstrating the expected time-dependent increase in false fear memory. This increase was comparable between PKMζ WT and PKMζ K281R mice. Additionally, freezing levels at 24 h in either overexpression group did not differ from those of controls (both *p* = 1.000; Figure 5H). Evaluation of the change in freezing (24–3 h) by two-way ANOVA yielded a significant US × Gene interaction [*F*(5,43) = 3.220, *p* < 0.05, η^2^_*p*_ = 0.27; Figure 5I]. However, post hoc comparisons showed that the magnitude of the increase in freezing in either PKMζ WT or PKMζ K281R mice was not significantly different from that of controls (both *p* = 1.000; Figure 5I). Together, these results indicated that the overexpression of either PKMζ WT or the kinase-inactive K281R mutant in the vHPC does not influence the formation or the time-dependent enhancement of false contextual fear memory.

### 3.6 The role of dorsal hippocampal PKMζ in hippocampus-dependent spatial memory

**FIGURE 6:**
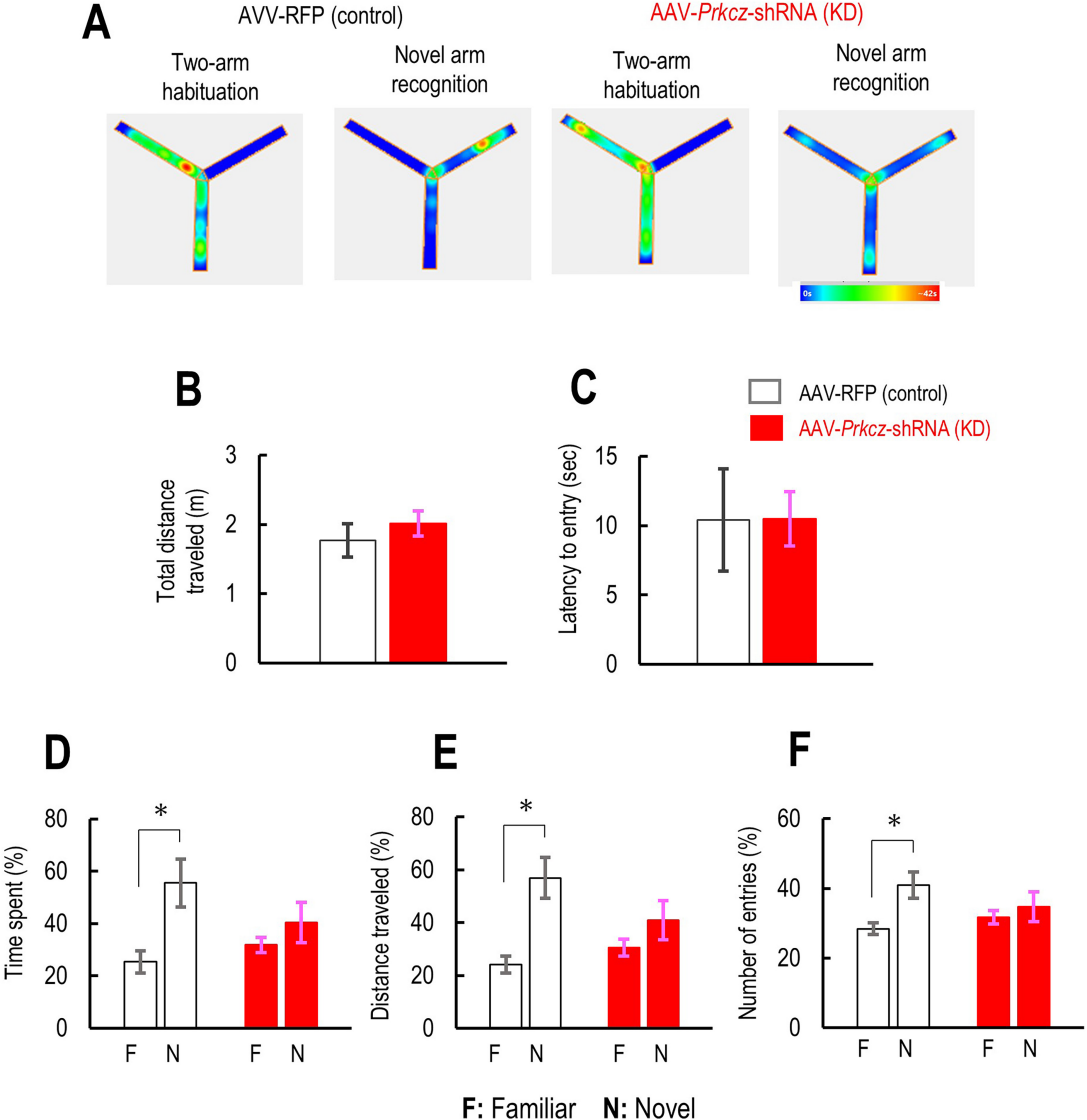
**(A)** Schematic of the Y-maze task and representative heat maps during the two-arm (training) and three-arm (test) phases. **(B)** Total distance traveled during the three-arm phase (control, *n* = 7; PKMζ KD, *n* = 8). **(C)** Latency to enter the novel arm (control, *n* = 7; PKMζ KD, *n* = 8). **(D)** Percentage of time spent in each arm during the three-arm phase (control, *n* = 7; PKMζ KD, *n* = 8). Values for the two familiar arms were averaged. **(E)** Percentage of total distance traveled in each arm during the three-arm phase (control, *n* = 7; PKMζ KD, *n* = 8). Values for the two familiar arms were averaged. **(F)** Number of entries into each arm during the three-arm phase (control, *n* = 7; PKMζ KD, *n* = 8). *p* < 0.05 (Student’s *t*-test). *p < 0.05, **p< 0.01.

To determine whether PKMζ in the vHPC contributes to hippocampus-dependent spatial memory, we evaluated the performance of vHPC PKMζ KD mice in the Y-maze novel arm recognition task. No difference in locomotor activity during the three-arm phase [*t*(12) = 0.8212, *p* = 0.427, Cohen’s *d* = 0.46; Figures 6A,B] or latency to enter the novel arm [*t*(12) = 1.670, *p* = 0.1209, Cohen’s *d* = 0.94; Figure 6C] was observed between control and PKMζ KD mice, indicating that general exploratory behavior remained unaffected. One-way ANOVA revealed significant group differences in both the time spent and the distance traveled in the arms [time: *F*(3,25) = 4.353, *p* < 0.05, η^2^_*p*_ = 0.34; distance: *F*(3,25) = 3.159, *p* < 0.05, η^2^_*p*_ = 0.28; Figures 6D, E]. Bonferroni-corrected *post hoc* comparisons showed that control mice spent significantly more time in, and traveled a greater distance within, the novel arm than in the familiar arms (both *p* < 0.05), demonstrating a normal novelty-directed preference. However, this preference was absent in PKMζ KD mice, which spent a similar amount of time and travelled a similar distance in both the novel and familiar arms. In addition, the number of entries into the novel arm differed significantly among groups [*F*(3,25) = 3.424, *p* = 0.035, η^2^_*p*_ = 0.29; Figure 6F], with PKMζ KD mice exhibiting fewer entries into the novel arm compared with control mice. Taken together, these findings indicated that PKMζ in the vHPC is required for hippocampus-dependent spatial memory and this requirement contrasts with the lack of involvement of vHPC PKMζ in the formation or time-dependent enhancement of false contextual fear memory.

## 4 Discussion

Evidence from mouse research indicates that false memories can form within 3 h of a fear experience, arising from rapid deterioration of the initially acquired fear memory (Lau et al., 2020). Consistent with this, false contextual fear responses in the current model have been shown to emerge rapidly within hours of training and undergo further strengthening upon re-exposure to a similar context (Kasama et al., 2022; Asano et al., 2025). In the present study, false fear was already detectable 3 h after conditioning when animals were placed in a context similar to the shock-paired environment (Context B). Re-exposure to Context B 24 h later produced a marked increase in freezing in animals previously exposed to the US, indicating that false contextual fear is not static but undergoes early, reactivation-dependent amplification. To identify the molecular substrates constraining this amplification, we selectively manipulated PKMζ, an atypical protein kinase C isoform implicated in synaptic plasticity and long-term memory maintenance (Sacktor, 2011; Patel and Zamani, 2021). Using AAV-PHP.eB-mediated gene delivery targeted predominantly to CA2/3 in adult mice (Mathiesen et al., 2020; Okamoto et al., 2023; Alexander et al., 2024), we performed three complementary manipulations. We found that the knockdown of PKMζ enhanced the increase in freezing between the 3 and 24-h time points, whereas the overexpression of wild-type PKMζ abolished this increase. Moreover, the overexpression of a kinase-inactive mutant (K281R) mimicked the knockdown phenotype, further enhancing re-exposure-dependent amplification. Importantly, none of these manipulations altered freezing at the 3-h time point, demonstrating that PKMζ is dispensable for the initial expression of false fear, but specifically regulates its strengthening following reactivation. Control US(−) animals showed minimal freezing across all time points, thereby excluding non-specific effects on baseline anxiety or locomotion. These bidirectional effects provide convergent evidence that PKMζ kinase activity in the dorsal CA2/CA3 functions as a molecular brake on the early amplification of false contextual fear.

Our previous work demonstrated that GR and MR signaling bidirectionally regulate false contextual fear memory (Asano et al., 2025). Specifically, we showed that the activation of GRs with dexamethasone facilitates false fear at the 3-h time point, whereas MR blockade with fludrocortisone prevents the enhancement observed at 24 h. These pharmacological effects are consistent with extensive evidence showing that GR activation disrupts hippocampal long-term potentiation (LTP) and impairs the retrieval of long-term spatial memory (Roozendaal et al., 2004; Cazakoff and Howland, 2010; Dorey et al., 2012; Shavit Stein et al., 2017; Oishi et al., 2019). In contrast, MR signaling supports synaptic plasticity and memory performance (Qiu et al., 2010; Maggio and Segal, 2012; Shavit Stein et al., 2017). The MR-enhanced LTP is not mediated by NMDA receptor activation, whereas GR-induced long-term depression (LTD) appears to be primarily NMDA-dependent. A growing body of evidence indicates that retrieval induces a transient destabilization of the memory trace, permitting its modification, attenuation, or enhancement before the updated memory is restabilized through reconsolidation (Kida, 2020; Cheng et al., 2022). In the present study, we found that PKMζ knockdown selectively enhanced false contextual fear at 24 h in Context B without altering its expression at 3 h, suggesting that PKMζ regulates post-retrieval strengthening rather than initial encoding. These findings raise the possibility that stress hormone receptor signaling and PKMζ-dependent maintenance mechanisms may jointly influence how early contextual overlap affects subsequent fear expression, although the present data do not establish a mechanistic link.

The novel arm recognition paradigm is widely used to assess spatial novelty detection, a hippocampus-dependent form of spatial recognition memory (Kraeuter et al., 2019). However, this assay does not directly measure contextual discrimination or representational precision. Therefore, the present data do not establish a causal relationship between spatial memory impairment and increased freezing in Context B. PKMζ has been classically associated with long-term memory maintenance. However, PKMζ inhibition disrupts LTP within 30–60 min after induction (Serrano et al., 2008; Sacktor, 2011), destabilizes the spatial firing stability of hippocampal place cells in a familiar environment (Barry et al., 2012), and facilitates memory destabilization while impairing reconsolidation (Chen et al., 2020). Moreover, prediction-error–induced exposure to a similar context can promote destabilization of the memory acquired in the original context (Chen et al., 2020), and such destabilization is accompanied by proteasome-dependent loss of synaptic PKMζ (Bernabo et al., 2021). These findings raise the possibility that the impairment of novel-arm recognition observed in the two-trial Y-maze test may involve a disruption of early PKMζ-dependent processes related to synaptic stabilization and the control of reactivation-induced destabilization. It is also possible that a reduction in PKMζ increases the susceptibility of reactivated contextual representations to destabilization, thereby influencing the stability of contextual representations and contributing to increased freezing in Context B. Future studies will be needed to clarify whether PKMζ regulates these processes during memory reactivation.

In interpreting the increased freezing observed in Context B, several considerations should be noted. First, the present study did not assess freezing in Context A at 3 or 24 h under PKMζ knockdown. This design choice was based on methodological considerations, as re-exposure to the conditioning context in the absence of the unconditioned stimulus can reactivate and modify the original memory trace through extinction- or reconsolidation-related processes (Kida, 2020; Cheng et al., 2022). Moreover, repeated exposure to Context A may promote encoding of the context as safe, thereby introducing extinction-like effects that complicate the interpretation of freezing behavior (Ouyang and Thomas, 2005). For this reason, testing was restricted to Context B to minimize such confounding influences. Nevertheless, the observed pattern of PKMζ-dependent freezing is consistent with reduced contextual memory precision. Our previous study using the same behavioral paradigm showed that early exposure to a similar context decreases freezing in Context A while increasing freezing in Context B, a bidirectional pattern that may reflect alterations in contextual representations rather than a simple increase in generalized fear (Asano et al., 2025). However, that study also included a condition in which mice were exposed to Context B only at 24 h. In that case, freezing levels at 24 h remained comparable to those observed at the 3 h time point, suggesting that early re-exposure may be necessary for the subsequent increase in freezing. The present design does not allow us to fully dissociate false contextual fear memory from fear generalization or interference-related processes. Because all animals were exposed to Context B at 3 h, the increased freezing observed at 24 h is compatible with multiple interpretations, including reduced contextual memory precision, generalization-like mechanisms, interference, or reconsolidation-dependent updating. The present findings are therefore consistent with several possible frameworks, and the specific contribution of early re-exposure cannot be determined in the absence of a condition in which Context B is presented only at 24 h.

In addition, the role of PKMζ in the dorsal hippocampus for contextual memory maintenance remains debated. Hippocampal PKMζ inhibition has been reported to impair spatial learning (Hsieh et al., 2021), whereas it has been reported that PKMζ inhibition in the hippocampus did not affect contextual information after fear conditioning (Serrano et al., 2008). Similarly, intra-hippocampal ZIP administration does not significantly affect the expression of established contextual fear memory under standard testing conditions, although clear effects are observed in other brain regions such as the amygdala, suggesting a region-dependent contribution of PKMζ to memory expression (Kwapis et al., 2009). On the other hand, knockdown of PKMζ disrupts established long-term memory, supporting a role for PKMζ in memory maintenance (Wang et al., 2016). The present study employed an interference-based paradigm, in which exposure to a similar context (Context B) occurs during an early post-conditioning phase.

A striking feature of false contextual fear memory in this model is its progressive, time-dependent escalation. Asano et al. (2025) found that freezing responses were stronger at 9 days than at 24 h after the initial fear experience when animals were exposed to a similar but distinct context shortly after conditioning. When mice were not exposed to Context B until 9 days after receiving an electric shock in Context A, the resulting false fear response did not differ significantly from that at 24 h (Asano et al., 2025). This indicates that false contextual fear is not merely a byproduct of imperfect encoding but a dynamically evolving process. Although several studies have documented false contextual fear memory, they have primarily focused on pre-exposure paradigms in which contextual memories formed prior to conditioning gradually become indistinguishable from the conditioning context (Ohkawa et al., 2015; Nomoto et al., 2023). Consequently, such paradigms do not allow investigation into how false fear memory is strengthened or the mechanisms that drive its time-dependent amplification. In contrast, our model captures the early, progressive escalation of false contextual fear, thereby providing a more suitable framework for elucidating the processes underlying its temporal strengthening. Studies have frequently conceptualized fear overgeneralization as a remote phenomenon emerging over weeks, during which memory specificity erodes as a result of systems consolidation or impaired pattern separation (Besnard and Sahay, 2016; Lopresto et al., 2016). Indeed, classical contextual fear paradigms show reduced context specificity at remote time points (14–28 days post-conditioning), coinciding with shifts in hippocampal–cortical dynamics (Germer et al., 2019; Nagayoshi et al., 2022). However, accumulating evidence indicates that generalization can also arise on much shorter timescales, particularly when similar or neutral contexts are encountered soon after conditioning (Leal et al., 2014; Fujinaka et al., 2016; Yu et al., 2021). These observations are consistent with the idea that generalization is not exclusively a remote phenomenon but can be initiated during early consolidation or reactivation phases. Our present findings extend this framework by demonstrating that false contextual fear can be further amplified upon early re-exposure to a similar context, and that this amplification is constrained by PKMζ activity in dorsal CA3. Importantly, PKMζ manipulations did not alter initial false fear expression at 3 h, but selectively modulated the increase observed at 24 h following re-exposure. These results suggest that PKMζ does not determine whether overlapping contextual representations are formed, but rather limits their reactivation-dependent strengthening.

Conceptually, the early amplification of false contextual fear may bear relevance to stress- and trauma-related conditions characterized by exaggerated responses to partially overlapping cues. In conditions such as PTSD, intrusive retrieval of trauma-associated representations can be triggered by stimuli that share contextual features with the original event, a process linked to the impairment of hippocampal contextual discrimination (Bisby and Burgess, 2013; Brewin, 2014; Smid et al., 2022). Although in the present study PTSD was not directly modeled, we identified a molecular mechanism in dorsal CA2/CA3 that constrains the early expansion of fear representations following reactivation. Such mechanisms may be associated with biological factors that limit maladaptive generalization under stress-related conditions. Since the present study was conducted exclusively in male mice, it remains to be determined whether PKMζ serves an equivalent role as a molecular brake in female mice. Women exhibit nearly twice the lifetime prevalence of PTSD compared to men, suggesting that sex-specific mechanisms may shape vulnerability to fear generalization (Hiscox et al., 2023). Future investigations should therefore incorporate sex as a biological variable, with particular attention to how gonadal hormones — such as estrogen — may modulate hippocampal pattern separation and the spatiotemporal dynamics of PKMζ activity (Taxier et al., 2020).

Several limitations should be considered. The present study did not assess freezing behavior in the original conditioning context (Context A) at later time points, as re-exposure to the conditioning context in the absence of the unconditioned stimulus is known to engage extinction- or reconsolidation-related processes that can modify the original memory trace (Kida, 2020; Cheng et al., 2022). In addition, prior exposure to a similar context may influence subsequent freezing responses, indicating that contextual experience can introduce interference effects (Ohkawa et al., 2015; Nomoto et al., 2023). Therefore, freezing behavior measured in Context A following repeated exposures may not serve as a straightforward index of memory retention, but instead reflects a combination of processes, including memory strength, extinction-like mechanisms, and reconsolidation-related modifications. Importantly, for this reason, our conclusions are based primarily on Context B–induced modulation of fear expression, which is interpreted as reflecting interference-related changes in contextual memory processing rather than direct alterations in Context A memory retention. To minimize these confounds, we employed the Y-maze task as a complementary approach to assess hippocampus-dependent spatial memory under non-stress conditions. However, this task does not directly assess contextual discrimination or memory precision, and thus the interpretation of reduced memory fidelity remains indirect. Further studies will be required to directly examine how PKMζ contributes to contextual memory precision and its relationship to time-dependent changes in freezing in similar contexts.

## Data Availability

The original contributions presented in the study are included in this article/Supplementary material, further inquiries can be directed to the corresponding authors.
