# Supplementary material for: Functional loss of PKMζ in the dorsal hippocampus potentiates the time-dependent increase in false contextual fear memory and impairs spatial recognition memory in mice
Source: Front Behav Neurosci. 2026 Jun 11;20:1837349. doi: 10.3389/fnbeh.2026.1837349 (PMC13294305; doi:10.3389/fnbeh.2026.1837349)
Supplement: Supplementary file 1 [file Table_1.docx]

Supplementary Material

# Supplementary Figure


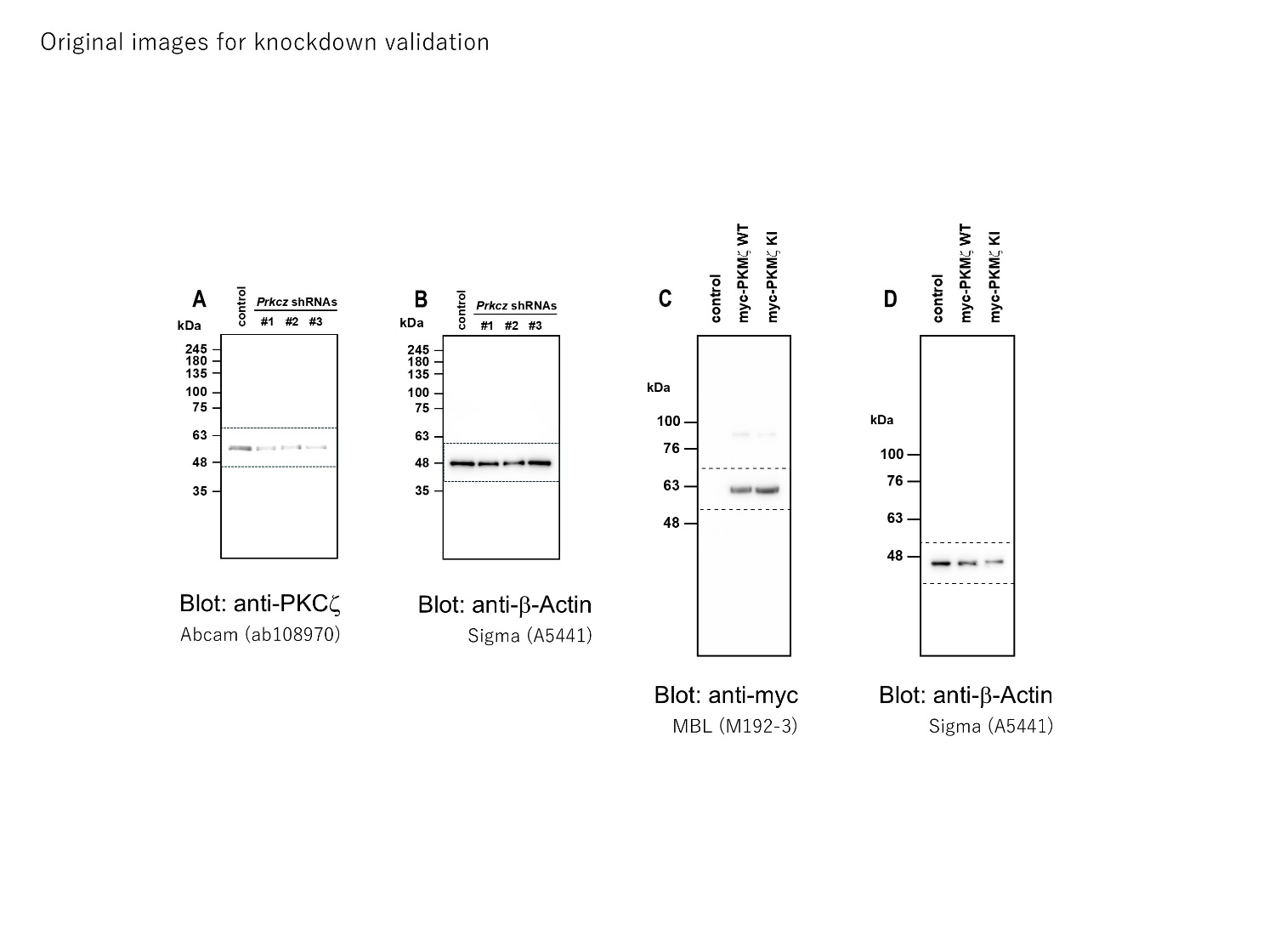


**Supplementary Figure 1.** **Original blots corresponding to the cropped images shown in Figure 1C and 2C.** (**A**) Full-length membrane images showing reduced PKMζ levels in primary cultured neurons transduced with AAV-*Prkcz* shRNA compared with those transduced with AAV-RFP, detected with an anti-PKMζ antibody (Abcam, ab108970). (**B**) Full-length membrane images showing β-actin expression in primary cultured neurons transduced with AAV-*Prkcz* shRNA or AAV-RFP, detected with an anti‑β-actin antibody (Sigma-Aldrich, A5441). (**C**) Full-length membrane images showing expressed PKMζ in primary cultured neurons transduced with AAV-PKMζ-WT or AAV-PKMζ-K281R, detected with an anti-c-Myc antibody (MBL, M192-3). (**D**) Full-length membrane images showing β-actin loading controls detected with an anti‑β‑actin antibody (Sigma-Aldrich, A5441).
